# Supplementary material for: Personalized functional brain network topography is associated with individual differences in youth cognition
Source: Nat Commun. 2023 Dec 18;14:8411. doi: 10.1038/s41467-023-44087-0 (PMC10728159; doi:10.1038/s41467-023-44087-0)
Supplement: Supplementary file 1 — Supplementary Information [file 41467_2023_44087_MOESM1_ESM.pdf]

Supplementary Information

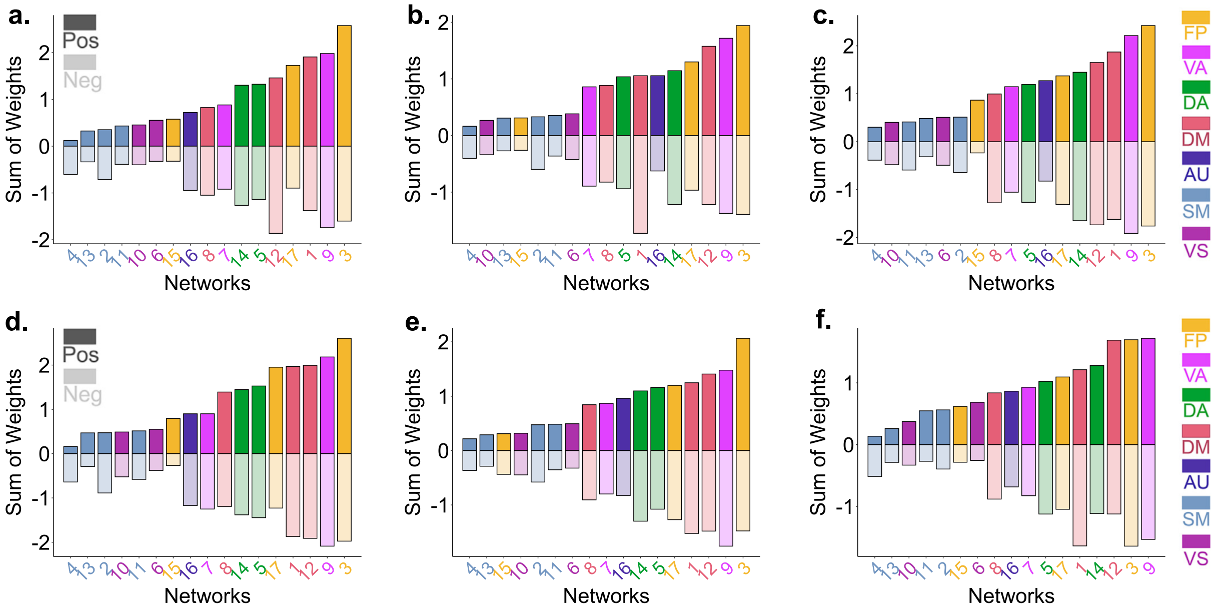

Supplementary Figure 1. Sum of ridge regression weights by PFN. Replication of results from Cui et al.[16] showing that the PFNs contributing the strongest positive (dark bars) and negative (light bars) weights in ridge regression models predicting cognition tend to lie at the associative end of the S-A axis. At each location on the cortex, the absolute contribution weight of each network was summed. **a,b,c** Models trained on the replication sample (n = 3,447) and tested in the matched discovery sample (n = 3,525); **d,e,f** Models trained on the discovery sample (n = 3,525) and tested in the matched replication sample (n = 3,447). **a,d** general cognition; **b,e** executive function; **c,f** learning/memory. (FP = Fronto-Parietal; VA = Ventral Attention; DA = Dorsal Attention; DM = Default Mode; AU = Auditory; SM = Somatomotor; VS = Visual).

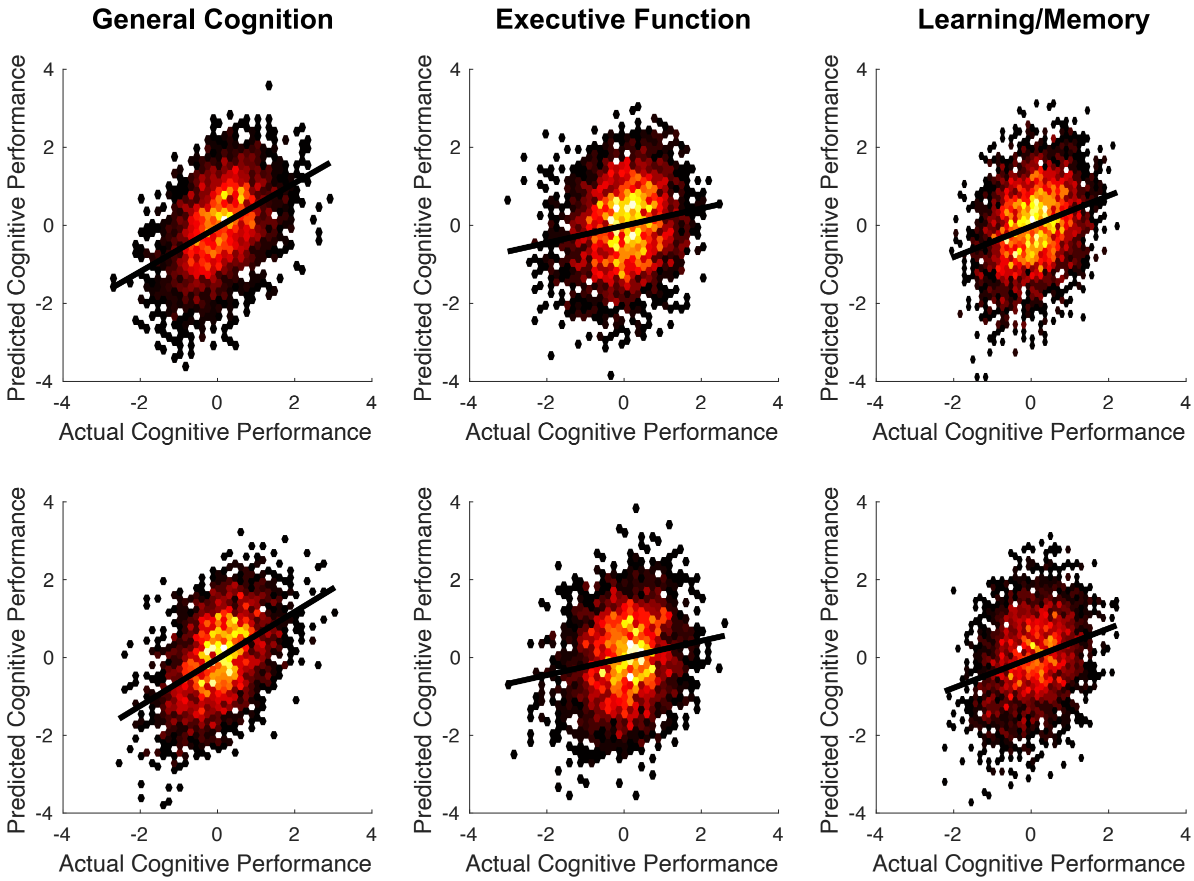

Supplementary Figure 2. Hexplots of associations between actual and predicted cognitive performance from ridge regression models. Association between actual and predicted cognitive performance using two-fold cross-validation (2F-CV) with nested cross-validation for parameter tuning across both the discovery (top row; *n* = 3,525) and replication (bottom row; *n* = 3,447) samples, for predictions of General Cognition (first column), Executive Function (second column), and Learning/Memory (third column). Heatmap represents the density of points plotted in a given region.

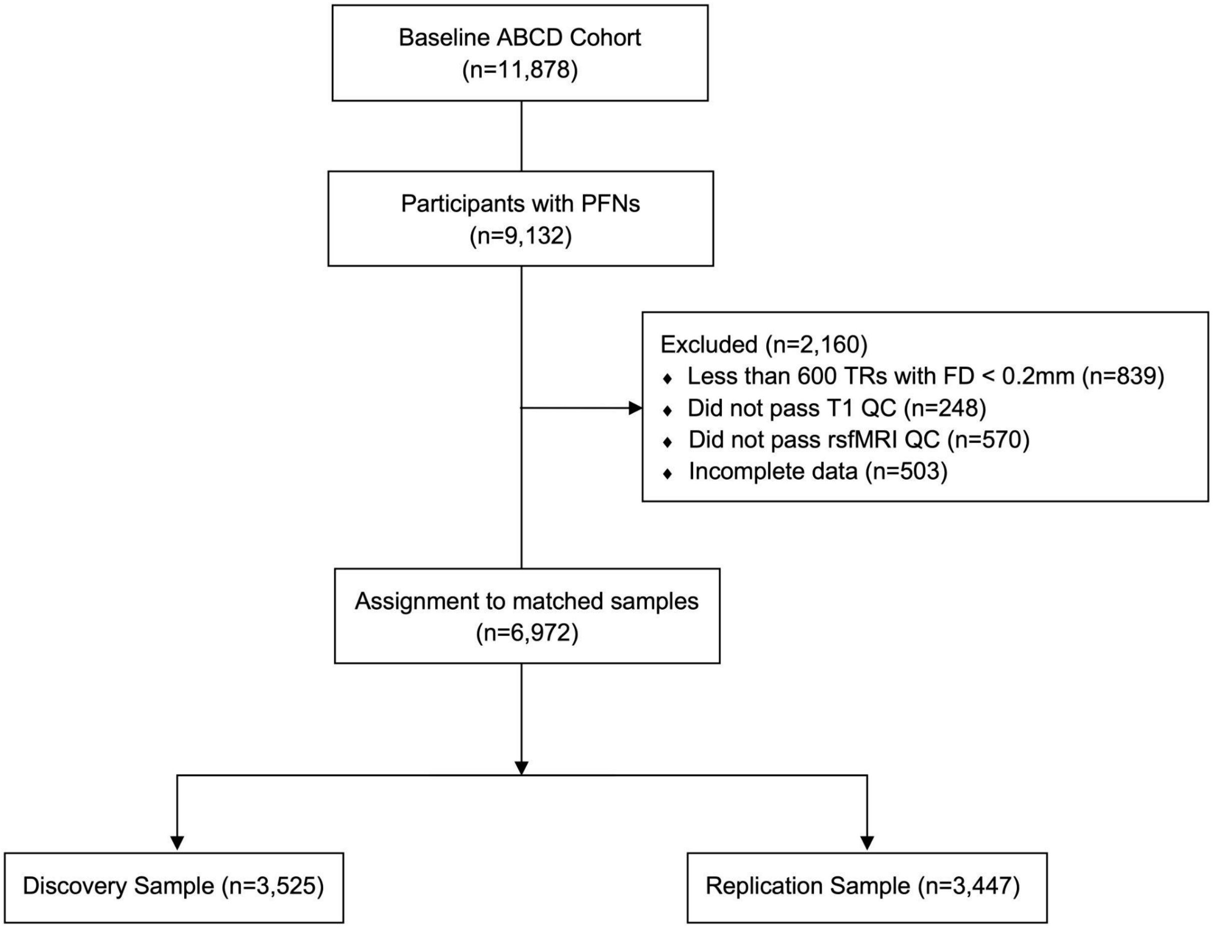

Supplementary Figure 3. Flow diagram depicting data inclusion and exclusion. Data were drawn from the Adolescent Brain and Cognitive Development (ABCD) study[39] baseline sample from the ABCD BIDS Community Collection (ABCC, ABCD-3165[40]), which included *n*=11,878 children between the ages of 9-11 years old. Participants were excluded for having incomplete data or excessive head motion, then split into a discovery sample (*n*=3,525) and a matched replication sample (*n*=3,447).[40,41]

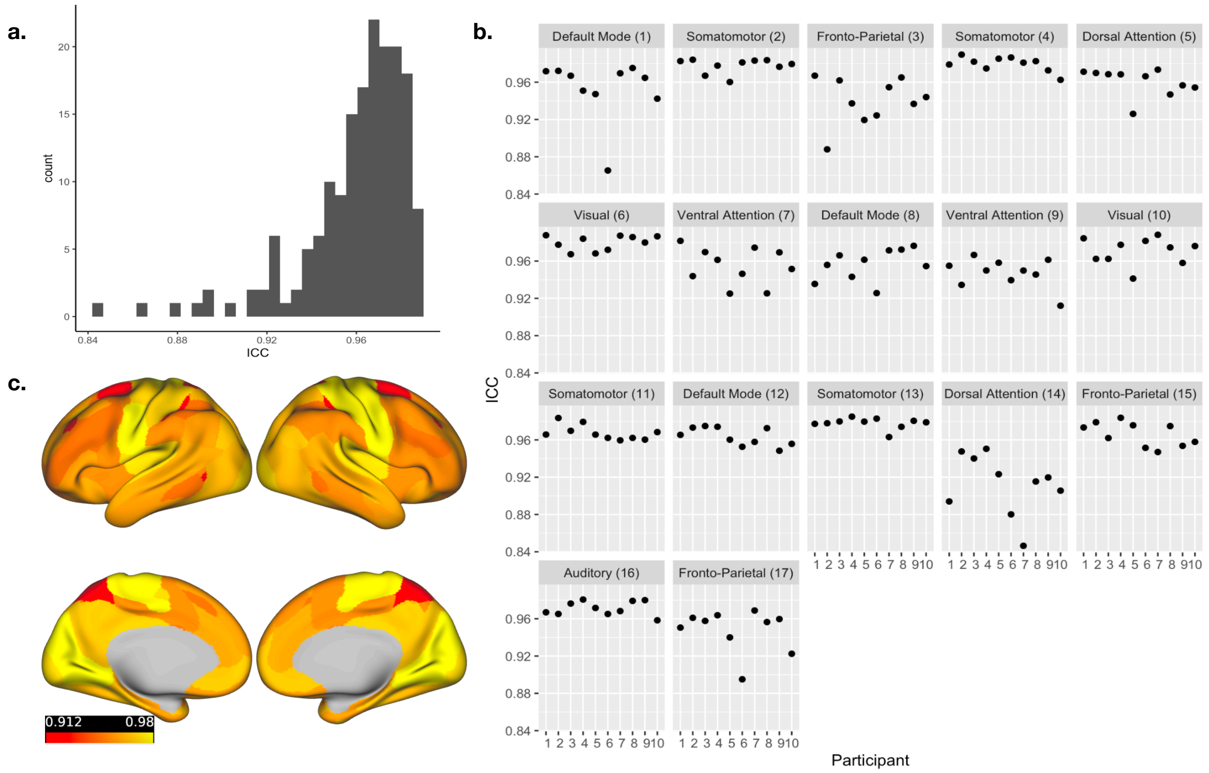

Supplementary Figure 4. Split-Half Reliability of PFN Topography. To assess split-half reliability of PFN functional topography, we conducted an additional analysis in which we leveraged data from *n* = 10 participants who underwent an additional resting-state scan. We first computed PFNs in each half of the data using the approach described in the main text and then calculated the split-half reliability for each participant and each PFN as the intraclass correlation coefficient (ICC) between the vertex-wise loadings in each half of the data. To contextualize our results: ICC scores greater than 0.9 are considered "excellent" while scores between 0.75 to 0.9 are considered "good", scores between 0.5 and 0.75 are considered "moderate" and scores below 0.5 are considered "poor" (Koo and Li, 2017). Across all seventeen PFNs and all ten participants, all ICC scores fell within a range of 0.84 to 0.99, with the majority of ICC scores (96%) falling in the "excellent" category (ICC > 0.9). ICC scores for each participant and each network are shown in (**a**) a histogram of all ICC values, (**b**) scatterplots depicting ICC scores for each of the ten participants by PFN, and (**c**) a brain map depicting average ICC score by network.

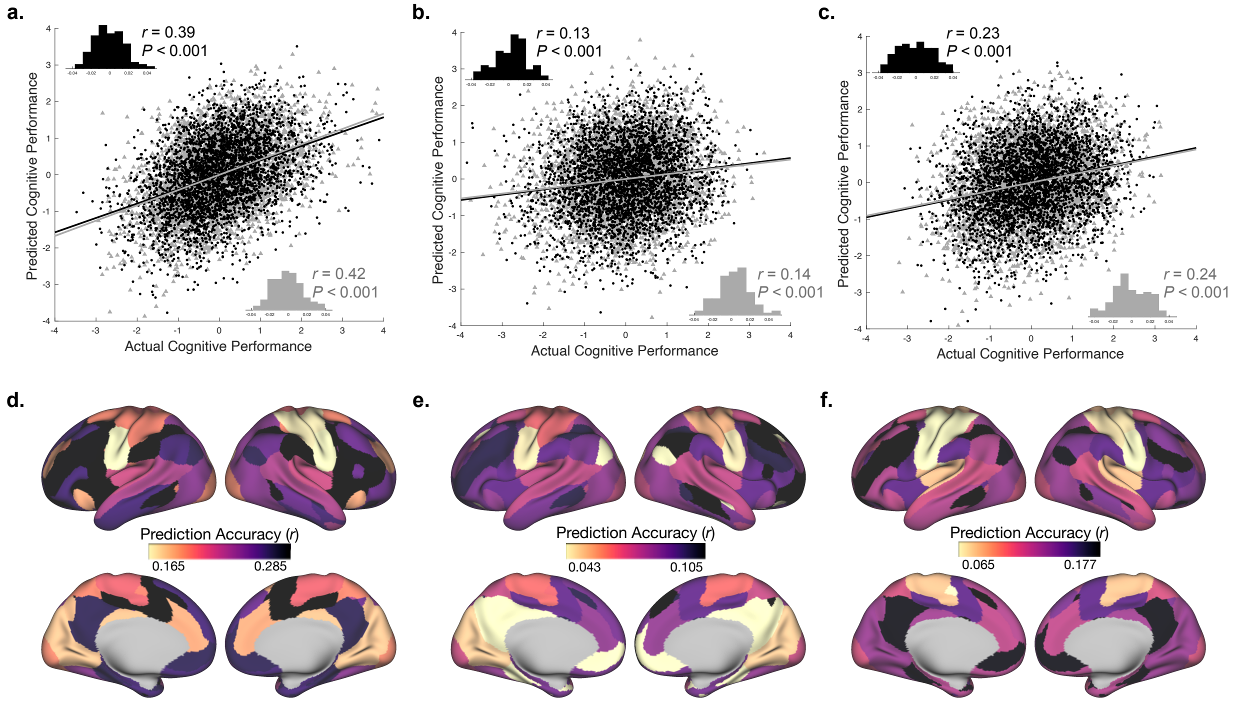

Supplementary Figure 5. Functional Topography Predicts Individual Differences in Cognitive Domains Derived by PCA in Independent Discovery and Replication Samples. To avoid contamination across discovery (n = 3,525) and replication (n = 3,447) samples in the cognitive outcome score that could lead to overfitting, we re-computed the cognitive domains of general cognition, executive function, and learning/memory using principal components analysis (PCA) conducted independently in the discovery and replication samples. Scatterplots depict the association between actual and predicted cognitive performance from ridge regression models trained to predict individual differences in general cognition (**a**), executive function (**b**), and learning/memory (**c**), using 2F-CV across both the discovery (black scatterplot) and replication (gray scatterplot) samples. Inset histograms represent the distributions of prediction accuracies from a permutation test. Brain maps depict the prediction accuracy results for ridge regression models trained to predict general cognition (**d**), executive function (**e**), or learning/memory (**f**) from the spatial topography of each PFN independently, with the highest prediction accuracies found in association cortex.

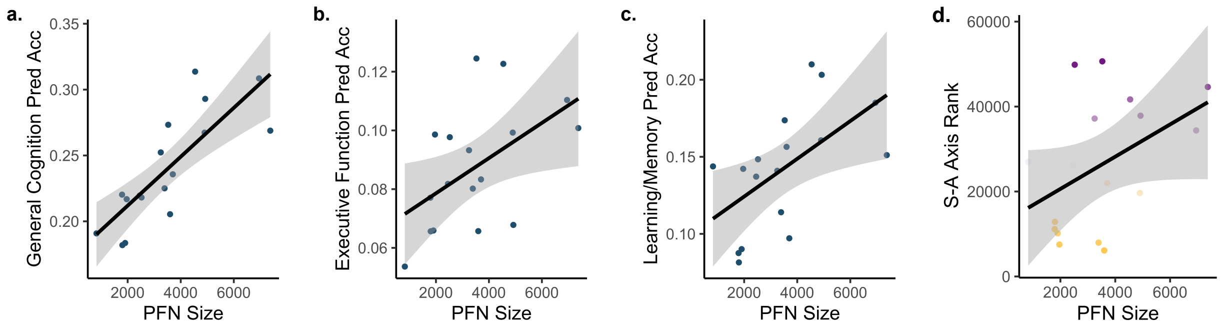

Supplementary Figure 6. Prediction accuracy and S-A axis rank by PFN size. Prediction accuracies by network (the average Pearson correlation *r* between actual and predicted cognitive performance across discovery (*n* = 3,525) and replication (*n* = 3,447) samples) from ridge regression models trained on the vertex-wise pattern of topography for each PFN to predict (**a**) General Cognition, (**b**) Executive Function, or (**c**) Learning/Memory are plotted on the y-axes. PFN sizes, defined as the number of vertices belonging to each PFN in the hard parcellation, are plotted on the x-axes. The correlations between PFN size and prediction accuracy are statistically significant for all three cognitive domains (General Cognition: $r(17) = 0.80$, $p < 0.001$; Executive Function: $r(17) = 0.52$, $p = 0.032$; Learning/Memory: $r(17) = 0.57$, $p = 0.017$). (**d**) Association between sensorimotor-association (S-A) axis rank and PFN size ($r(17) = 0.44$, $p = 0.077$).

	Discovery		Replication	
Prediction Accuracy	*r*	*p*	*r*	*p*
**General Cognition**				
SES	0.26	1.35×10^{-51}	0.28	4.77×10^{-58}
PFN Topography	0.41	3.05×10^{-146}	0.45	3.85×10^{-174}
SES + PFN Topography	0.43	1.01×10^{-151}	0.46	1.80×10^{-171}
**Executive Function**				
SES	0.07	1.14×10^{-4}	0.09	3.25×10^{-7}
PFN Topography	0.17	1.37×10^{-23}	0.16	5.48×10^{-22}
SES + PFN Topography	0.17	7.18×10^{-22}	0.17	2.59×10^{-23}
**Learning/Memory**				
SES	0.13	2.96×10^{-13}	0.16	4.46×10^{-19}
PFN Topography	0.27	2.06×10^{-61}	0.27	2.91×10^{-57}
SES + PFN Topography	0.27	3.53×10^{-57}	0.27	2.35×10^{-56}

Supplementary Table 1. Predictive models incorporating socio-economic status (SES). Prediction accuracy, measured as the Pearson correlation *r* between actual and predicted cognitive performance with raw *p*-values, is shown for ridge regression models trained to predict cognitive performance across three domains (General Cognition, Executive Function, and Learning/Memory) across both discovery and replication samples. Results from three sets of predictive models are shown: "SES" refers to models trained only on socio-economic status as measured by the areal deprivation index; "PFN Topography" refers to models trained on the multivariate pattern of personalized functional brain network (PFN) topography for each individual (as presented in the main text and in Figures 2 and 3); and "SES + PFN Topography" refers to models trained on both socio-economic status and PFN topography. Although SES is a significant predictor of cognitive functioning, models trained on PFN topography yield much stronger predictions of cognitive performance than SES alone, and the addition of SES information to models trained on PFN topography does not substantially increase prediction accuracy. This observation suggests that the spatial topography of individually-defined functional brain networks accounts for additional inter-individual variance in cognitive performance beyond what is accounted for by SES alone.

	PFN 3				PFN 15				PFN 17			
Predictors	*β*	*Std. Error*	*t*	p_{bonf}	*β*	*Std. Error*	*t*	p_{bonf}	*β*	*Std. Error*	*t*	p_{bonf}
**Discovery**												
Intercept	0.49	0.22	2.20	**0.028**	-0.10	0.22	-0.44	0.659	0.18	0.23	0.79	0.428
Age	-0.05	0.02	-2.62	**0.009**	-0.02	0.02	-1.27	0.206	-0.05	0.02	-2.62	**0.009**
Sex	-0.06	0.03	-1.78	0.075	-0.15	0.03	-4.53	$\mathbf{6.17 \times 10^{-6}}$	0.05	0.03	1.36	0.174
Mean FD	0.12	0.02	7.02	$\mathbf{2.56 \times 10^{-12}}$	0.11	0.02	6.78	$\mathbf{1.38 \times 10^{-11}}$	0.04	0.02	2.25	**0.025**
ADHD Meds	-0.04	0.07	-0.53	0.594	-0.05	0.07	-0.81	0.419	-0.08	0.07	-1.20	0.231
Antipsychotic Meds	-0.36	0.22	-1.65	0.099	0.22	0.21	1.03	0.304	-0.24	0.22	-1.11	0.267
Antidepressant Meds	-0.09	0.13	-0.71	0.475	-0.00	0.13	-0.01	0.991	0.10	0.13	0.80	0.421
General Cognition	0.08	0.02	3.41	$\mathbf{6.69 \times 10^{-4}}$	0.09	0.02	3.49	$\mathbf{4.87 \times 10^{-4}}$	0.11	0.02	4.41	$\mathbf{1.07 \times 10^{-5}}$
**Replication**												
Intercept	0.04	0.26	0.16	0.871	-0.18	0.26	-0.68	0.498	0.12	0.26	0.47	0.637
Age	-0.01	0.02	-0.81	0.418	-0.06	0.02	-3.29	**0.001**	-0.05	0.02	-2.68	**0.007**
Sex	-0.04	0.03	-1.16	0.245	-0.15	0.03	-4.18	$\mathbf{2.94 \times 10^{-5}}$	0.04	0.03	1.06	0.287
Mean FD	0.15	0.02	8.98	$\mathbf{4.27 \times 10^{-19}}$	0.08	0.02	4.53	$\mathbf{6.19 \times 10^{-6}}$	0.04	0.02	2.11	**0.035**
ADHD Meds	0.12	0.07	1.82	0.069	0.02	0.07	0.27	0.791	-0.01	0.07	-0.13	0.896
Antipsychotic Meds	-0.15	0.25	-0.62	0.534	0.34	0.24	1.40	0.163	0.06	0.25	0.23	0.817
Antidepressant Meds	0.00	0.14	0.03	0.977	-0.11	0.14	-0.80	0.423	-0.20	0.14	-1.41	0.159
General Cognition	0.07	0.02	2.97	**0.003**	0.09	0.03	3.65	$\mathbf{2.63 \times 10^{-4}}$	0.12	0.02	4.70	$\mathbf{2.66 \times 10^{-6}}$

Supplementary Table 2. Sensitivity analyses controlling for psychotropic medication use. Linear mixed effects models associating general cognition with the total cortical representation of fronto-parietal PFNs remain significant across both the discovery and replication samples when controlling for psychotropic medication use (assessed by the Medication Inventory from the PhenX instrument and coded as in Shoval et al., 2021; though we note that it is not clear from these measures whether psychotropic medications were taken on the same day as the neuroimaging assessments). *P*-values are corrected for multiple comparisons using Bonferroni correction.

	PFN 3				PFN 15				PFN 17			
Predictors	*β*	*Std. Error*	*t*	p_{bonf}	*β*	*Std. Error*	*t*	p_{bonf}	*β*	*Std. Error*	*t*	p_{bonf}
**Discovery**												
Intercept	0.01	0.02	0.42	0.672	0.08	0.02	3.24	**1.21 x 10^{-3}**	-0.04	0.02	-1.80	7.26e-02
Age	-0.04	0.02	-2.23	**0.026**	-0.03	0.02	-1.43	0.152	-0.05	0.02	-2.59	**9.65 x 10^{-3}**
Sex	-0.05	0.04	-1.51	0.131	-0.16	0.03	-4.53	**6.23 x 10^{-6}**	0.07	0.03	1.91	0.0557
Mean FD	0.12	0.02	7.07	**1.83 x 10^{-12}**	0.12	0.02	6.65	**3.36 x 10^{-11}**	0.04	0.02	2.08	**0.038**
SES	-0.00	0.02	-0.14	0.892	0.01	0.02	0.51	0.610	0.02	0.02	1.34	0.179
General Cognition	0.08	0.03	3.20	**1.40 x 10^{-3}**	0.08	0.03	3.10	**1.96 x 10^{-3}**	0.11	0.03	4.06	**5.09 x 10^{-5}**
**Replication**												
Intercept	0.01	0.03	0.44	0.661	0.07	0.03	2.62	**8.86 x 10^{-3}**	-0.02	0.03	-0.78	0.436
Age	-0.02	0.02	-0.86	0.392	-0.06	0.02	-3.13	**1.77 x 10^{-3}**	-0.05	0.02	-2.68	**7.35 x 10^{-3}**
Sex	-0.05	0.04	-1.43	0.154	-0.15	0.04	-4.10	**4.22 x 10^{-5}**	0.03	0.04	0.86	0.388
Mean FD	0.16	0.02	9.32	**2.18 x 10^{-20}**	0.09	0.02	4.84	**1.37 x 10^{-6}**	0.04	0.02	2.41	**0.016**
SES	0.03	0.02	1.50	0.133	0.05	0.02	2.71	**6.71 x 10^{-3}**	0.04	0.02	2.36	**0.018**
General Cognition	0.07	0.03	2.53	**0.011**	0.07	0.03	2.57	**0.010**	0.10	0.03	3.93	**8.51 x 10^{-5}**

Supplementary Table 3. **Sensitivity analyses controlling for socio-economic status (SES).** Linear mixed effects models associating general cognition with the total cortical representation of fronto-parietal PFNs remain significant across both the discovery and replication samples when controlling for socio-economic status (SES) as measured by areal deprivation index. *P*-values are corrected for multiple comparisons using Bonferroni correction.

a. Univariate Association Results

Predictors	PFN 3				PFN 15				PFN 17			
	β	*Std. Error*	*t*	p_{bonf}	*β*	*Std. Error*	*t*	p_{bonf}	*β*	*Std. Error*	*t*	p_{bonf}
**Discovery**												
Intercept	0.02	0.03	0.71	0.476	0.07	0.03	2.48	**0.013**	-0.03	0.03	-1.06	0.289
Age	0.01	0.02	0.28	0.778	-0.02	0.02	-0.86	0.388	-0.04	0.02	-1.84	0.065
Sex	-0.07	0.04	-1.77	0.076	-0.16	0.04	-4.10	**4.26×10^{-5}**	0.05	0.04	1.30	0.195
Mean FD	-0.13	0.02	-6.71	**2.42×10^{-11}**	0.17	0.02	8.67	**7.13×10^{-18}**	-0.11	0.02	-5.57	**2.84×10^{-8}**
General Cognition	0.17	0.03	6.01	**2.16×10^{-9}**	0.05	0.03	1.79	0.074	0.08	0.03	2.97	**0.003**
**Replication**												
Intercept	0.04	0.03	1.33	0.183	0.05	0.03	1.96	0.051	0.01	0.03	0.32	0.747
Age	-0.01	0.02	-0.45	0.649	-0.02	0.02	-0.99	0.323	-0.01	0.02	-0.46	0.646
Sex	-0.08	0.04	-2.24	**0.025**	-0.13	0.04	-3.37	**7.65×10^{-4}**	-0.01	0.04	-0.15	0.881
Mean FD	-0.14	0.02	-7.78	**1.01×10^{-14}**	0.14	0.02	7.67	**2.36×10^{-14}**	-0.05	0.02	-2.76	**0.006**
General Cognition	0.21	0.03	8.01	**1.60×10^{-15}**	0.10	0.03	3.76	**1.74×10^{-4}**	0.08	0.03	2.85	**0.004**

b. Multivariate Prediction Results

Prediction Accuracy	Discovery		Replication	
	r	*p*	*r*	*p*
**General Cognition**	0.41	7.96×10^{-108}	0.41	5.62×10^{-115}
**Executive Function**	0.16	2.63×10^{-16}	0.12	3.02×10^{-11}
**Learning/Memory**	0.26	7.38×10^{-43}	0.25	2.47×10^{-40}

Supplementary Table 4. Replication of our main analyses using PFNs derived from resting-state data only. (**a**) Univariate association analyses using linear mixed effects models confirm that the total cortical representation of fronto-parietal PFNs 3, 15 and 17 are positively associated with general cognition across the discovery and replication samples. *P*-values are corrected for multiple comparisons using Bonferroni correction. (**b**) Multivariate prediction results from ridge regression models trained to predict cognitive performance from the multivariate pattern of PFN topography show comparable prediction accuracy (the Pearson correlation *r* between actual and predicted cognitive performance with raw *p*-values) as in our main results for both the discovery and replication samples. Results are depicted for *n*=5,968 participants who had sufficient TRs remaining in the rest-only data.

	Discovery		Replication	
Prediction Accuracy	*r*	*p*	*r*	*p*
**Picture Vocabulary**	0.38	7.25×10^{-120}	0.40	4.55×10^{-132}
**Oral Reading Test**	0.34	3.39×10^{-93}	0.39	3.66×10^{-127}
**List Sort**	0.29	2.54×10^{-69}	0.32	5.87×10^{-82}
**RAVLT**	0.28	1.34×10^{-63}	0.28	4.18×10^{-64}
**LMT**	0.24	3.76×10^{-46}	0.24	1.93×10^{-47}
**Card Sorting**	0.22	9.24×10^{-39}	0.22	7.36×10^{-38}
**Picture Sequence**	0.20	1.85×10^{-31}	0.19	8.13×10^{-29}
**Flanker Test**	0.13	9.11×10^{-15}	0.13	5.54×10^{-14}
**Pattern Comparison**	0.11	3.06×10^{-10}	0.12	4.76×10^{-12}

Supplementary Table 5. Prediction accuracy of ridge regression models predicting performance on individual cognitive tasks. Multivariate prediction results from ridge regression models trained to predict cognitive performance from the multivariate pattern of PFN topography show comparable prediction accuracies (the Pearson correlation *r* between actual and predicted cognitive performance with raw *p*-values) as in our main results using PCA scores: the highest prediction accuracies are found for the top two tasks that loaded most highly on the first principal component of General Cognition, while the lowest prediction accuracies were found for the top two tasks loading highest on the second principal component of Executive Function and the two tasks loading highest on the third principal component of Learning/Memory fell in the middle across both the discovery and replication samples.
