# Supplementary material for: Personalized functional brain network topography is associated with individual differences in youth cognition
Source: Nat Commun. 2023 Dec 18;14:8411. doi: 10.1038/s41467-023-44087-0 (PMC10728159; doi:10.1038/s41467-023-44087-0)
Supplement: Supplementary file 3 — Reporting Summary [file 41467_2023_44087_MOESM3_ESM.pdf]

nature portfolio

Corresponding author(s): Theodore D. Satterthwaite
Last updated by author(s): Oct 22, 2023

Reporting Summary

Nature Portfolio wishes to improve the reproducibility of the work that we publish. This form provides structure for consistency and transparency in reporting. For further information on Nature Portfolio policies, see our Editorial Policies and the Editorial Policy Checklist.

Statistics

For all statistical analyses, confirm that the following items are present in the figure legend, table legend, main text, or Methods section.

n/a	Confirmed	
☐	☒	The exact sample size (*n*) for each experimental group/condition, given as a discrete number and unit of measurement
☐	☒	A statement on whether measurements were taken from distinct samples or whether the same sample was measured repeatedly
☐	☒	The statistical test(s) used AND whether they are one- or two-sided<br>*Only common tests should be described solely by name; describe more complex techniques in the Methods section.*
☐	☒	A description of all covariates tested
☐	☒	A description of any assumptions or corrections, such as tests of normality and adjustment for multiple comparisons
☐	☒	A full description of the statistical parameters including central tendency (e.g. means) or other basic estimates (e.g. regression coefficient) AND variation (e.g. standard deviation) or associated estimates of uncertainty (e.g. confidence intervals)
☐	☒	For null hypothesis testing, the test statistic (e.g. *F*, *t*, *r*) with confidence intervals, effect sizes, degrees of freedom and *P* value noted<br>*Give P values as exact values whenever suitable.*
☒	☐	For Bayesian analysis, information on the choice of priors and Markov chain Monte Carlo settings
☒	☐	For hierarchical and complex designs, identification of the appropriate level for tests and full reporting of outcomes
☐	☒	Estimates of effect sizes (e.g. Cohen's *d*, Pearson's *r*), indicating how they were calculated

Our web collection on statistics for biologists contains articles on many of the points above.

Software and code

Policy information about availability of computer code

Data collection	No software was used for data collection.
Data analysis	Data analysis was performed using Matlab (R2022a), R (4.1.3) and Python (3.9). Code is available at https://github.com/PennLINC/keller-networks.

For manuscripts utilizing custom algorithms or software that are central to the research but not yet described in published literature, software must be made available to editors and reviewers. We strongly encourage code deposition in a community repository (e.g. GitHub). See the Nature Portfolio guidelines for submitting code & software for further information.

Data

Policy information about availability of data

All manuscripts must include a data availability statement. This statement should provide the following information, where applicable:

- Accession codes, unique identifiers, or web links for publicly available datasets
- A description of any restrictions on data availability
- For clinical datasets or third party data, please ensure that the statement adheres to our policy

Data used in the preparation of this article were obtained from the Adolescent Brain Cognitive Development (ABCD) Study (https://abcdstudy.org), held in the NIMH Data Archive (NDA).

Research involving human participants, their data, or biological material

Policy information about studies with human participants or human data. See also policy information about sex, gender (identity/presentation), and sexual orientation and race, ethnicity and racism.

Reporting on sex and gender	Biological sex (self reported sex assigned at birth) was used as a covariate in all statistical models.
Reporting on race, ethnicity, or other socially relevant groupings	We used areal deprivation index (ADI) as a measure of socio-economic status in our analyses, given that socio-economic status has been associated with better performance on a wide range of cognitive tasks in prior work (Gonzalez et al., 2020; Kirlic et al., 2021; R. C. Thompson et al., 2022; Tomasi & Volkow, 2021). We have also included self-reported racial identity in our demographics table (Table 2) for each sub-sample of participants in our study.
Population characteristics	Demographic information regarding the sample of participants (9-10 year old children) used in this study can be found in Table 2.
Recruitment	Data were drawn from the Adolescent Brain Cognitive Development (ABCD) study baseline sample from the ABCD BIDS Community Collection (ABCC, ABCD-3165), which included children aged 9-10 years old and their parents/guardians collected across 21 sites in the United States. Inclusion criteria for this study included being within the desired age range (9-10 years old), English language proficiency in the children, and having the ability to provide informed consent (parent) and assent (child).
Ethics oversight	Institutional Review Board (IRB) approval was received from the University of California, San Diego and the respective IRBs of each study site.

Note that full information on the approval of the study protocol must also be provided in the manuscript.

Field-specific reporting

Please select the one below that is the best fit for your research. If you are not sure, read the appropriate sections before making your selection.

☐ Life sciences ☒ Behavioural & social sciences ☐ Ecological, evolutionary & environmental sciences

For a reference copy of the document with all sections, see nature.com/documents/nr-reporting-summary-flat.pdf

Behavioural & social sciences study design

All studies must disclose on these points even when the disclosure is negative.

Study description	Data used in the preparation of this article were obtained from the Adolescent Brain Cognitive Development (ABCD) Study (https://abcdstudy.org). This is a longitudinal study of brain development and child health collected from multiple sites across the United States, from which we drew data from the baseline assessment.
Research sample	Demographic information regarding the sample of participants (9-10 year old children) used in this study can be found in Table 2. We used over three thousand participants in each of our matched Discovery and Replication subsamples, ensuring we far exceeded the minimum number of participants needed for statistical power. The rationale for our choice to use this study sample includes the availability of sufficiently long duration functional magnetic resonance imaging scans, large sample size, breadth of cognitive assessments, and appropriate age range for studying children on the precipice of the transition to adolescence.
Sampling strategy	Data were drawn from the Adolescent Brain Cognitive Development (ABCD) study baseline sample from the ABCD BIDS Community Collection (ABCC, ABCD-3165), which included children aged 9-10 years old and their parents/guardians collected across 21 sites in the United States. Inclusion criteria for this study included being within the desired age range (9-10 years old), English language proficiency in the children, and having the ability to provide informed consent (parent) and assent (child). The sampling procedure for the ABCD study, which took place across 21 sites across the United States, has been described in detail (Garavan et al., 2018). No sample size calculation was performed for the present study, and the maximal number of ABCD Study participants with available, high-quality data were included. Our sample size was n=6,972, which according to prior work (Marek*,Tervo-Clemmens* et al., 2022) is more than sufficient for the statistical power required.
Data collection	Participants completed a battery of cognitive assessments, including seven tasks from the NIH Toolbox (Picture Vocabulary, Flanker Test, List Sort Working Memory Task, Dimensional Change Card Sort Task, Pattern Comparison Processing Speed Task, Picture Sequence Memory Task, and the Oral Reading Test) as well as two additional tasks (the Little Man Task and the Rey Auditory Verbal Learning Task). Neuroimaging acquisition for the ABCD study has been described previously (Casey et al., 2018).
Timing	ABCD data for the baseline assessment period that were used in this study were collected between 09/09/2016 and 10/15/2018.
Data exclusions	Exclusion criteria included the presence of severe sensory, intellectual, medical or neurological issues that would have impacted the child's ability to comply with the study protocol, as well as MRI scanner contraindications. As depicted in Supplementary Figure 1, we additionally excluded participants with incomplete data or excessive head motion, yielding a final sample of n= 6,972.
Non-participation	N/A

Randomization	Participants were not allocated into experimental groups.

Reporting for specific materials, systems and methods

We require information from authors about some types of materials, experimental systems and methods used in many studies. Here, indicate whether each material, system or method listed is relevant to your study. If you are not sure if a list item applies to your research, read the appropriate section before selecting a response.

Materials & experimental systems

n/a	Involved in the study
☒	☐ Antibodies
☒	☐ Eukaryotic cell lines
☒	☐ Palaeontology and archaeology
☒	☐ Animals and other organisms
☒	☐ Clinical data
☒	☐ Dual use research of concern
☒	☐ Plants

Methods

n/a	Involved in the study
☒	☐ ChIP-seq
☒	☐ Flow cytometry
☐	☒ MRI-based neuroimaging

Magnetic resonance imaging

Experimental design

Design type	Following preprocessing, we concatenated the time series data for both resting-state scans and three task-based scans (Monetary Incentive Delay Task, Stop-Signal Task, and Emotional N-Back Task) as in prior work to maximize the available data for our analyses. We then applied spatially-regularized non-negative matrix factorization to derive personalized functional brain networks and calculated the total spatial representation of each of these networks across the cortex.
Design specifications	N/A
Behavioral performance measures	No behavioral measures collected during fMRI scanning were used in this study.

Acquisition

Imaging type(s)	functional
Field strength	3.0
Sequence & imaging parameters	All information on sequence and imaging parameters for each type of scan from the ABCD study can be found in Casey et al. (2018), Developmental Cognitive Neuroscience.
Area of acquisition	Whole brain

Diffusion MRI ☐ Used ☒ Not used

Preprocessing

Preprocessing software	As previously described, the ABCC Collection 3165 from which we drew our data was processed according to the ABCD-BIDS pipeline. This pipeline includes distortion correction and alignment, denoising with Advanced Normalization Tools (ANTS73), FreeSurfer74 segmentation, surface registration, and volume registration using FSL FLIRT rigid-body transformation according to the DCAN BOLD Processing (DBP) pipeline which included the following steps: 1) de-meaning and de-trending of all fMRI data with respect to time; 2) denoising using a general linear model with regressors for signal and movement; 3) bandpass filtering between 0.008 and 0.09 Hz using a 2nd order Butterworth filter; 4) applying the DBP respiratory motion filter (18.582 to 25.726 breaths per minute), and 5) applying DBP motion censoring (frames exceeding an FD threshold of 0.2mm or failing to pass outlier detection at +/- 3 standard deviations were discarded).
Normalization	N/A
Normalization template	N/A
Noise and artifact removal	As previously described, the ABCC Collection 3165 from which we drew our data was processed according to the ABCD-BIDS pipeline. This pipeline includes distortion correction and alignment, denoising with Advanced Normalization Tools (ANTS73), FreeSurfer74 segmentation, surface registration, and volume registration using FSL FLIRT rigid-body transformation according to the DCAN BOLD Processing (DBP) pipeline which included the following steps: 1) de-meaning and de-trending of all fMRI data with respect to time; 2) denoising using a general linear model with regressors for signal and movement; 3) bandpass filtering between 0.008 and 0.09 Hz using a 2nd order Butterworth filter; 4) applying the DBP respiratory motion filter

(18.582 to 25.726 breaths per minute), and 5) applying DBP motion censoring (frames exceeding an FD threshold of 0.2mm or failing to pass outlier detection at +/- 3 standard deviations were discarded).

Volume censoring

Volumes were censored if they exceeded an FD threshold of 0.2mm using DBP motion censoring.

Statistical modeling & inference

Model type and settings

Our primary analyses used ridge regression models to predict cognitive performance in held-out children's data from their personalized functional brain network topography. Details of our multivariate modeling and predictive analysis may be found below.

Effect(s) tested

We used linear mixed effects models to assess associations between PFN topography and performance in each cognitive domain while accounting for both fixed and random predictors. All models included fixed effects parameters for age, biological sex, head motion (mean fractional displacement), as well as random intercepts for family (accounting for siblings) and site groupings.

Specify type of analysis: ☒ Whole brain ☐ ROI-based ☐ Both

Statistic type for inference

(See Eklund et al. 2016)

N/A

Correction

multiple comparisons were accounted for using the Bonferroni method

Models & analysis

n/a	Involved in the study
☒	☐ Functional and/or effective connectivity
☒	☐ Graph analysis
☐	☒ Multivariate modeling or predictive analysis

Multivariate modeling and predictive analysis

We used non-negative matrix factorization (NMF) to derive individualized functional networks. NMF identifies networks by positively weighting connectivity patterns that covary, leading to a highly specific and reproducible parts-based representation. Using the previously-derived group consensus atlas as a prior to ensure inter-individual correspondence, we derived each individual's specific network atlas using NMF based on the acquired group networks as initialization and each individual's specific fMRI times series. This procedure yielded a loading matrix for each participant, where each row is a personalized functional brain network, each column is a vertex, and the value quantifies the extent each vertex belongs to each network. We trained ridge regression models to predict cognitive performance in each of the three cognitive domains (general cognition, executive function, and learning/memory) using the functional topography (vertex-wise network loading matrices) of each participant's PFNs. Models were trained to predict cognitive performance in held-out children's data from concatenated network loading matrices across the 17 PFNs. Independent network models were also trained on the network-wise loadings at each vertex. All models included covariates for age, sex, site, and motion (mean FD). Our primary ridge regression models were trained and tested on the ABCD reproducible matched samples using two-fold cross-validation (2F-CV). To ensure that this sample selection procedure did not bias our results, we performed repeated random cross-validation over 100 iterations, each time randomly splitting the sample and repeating the 2F-CV procedure to generate a distribution of prediction accuracies for each model. Furthermore, we used permutation testing to generate null distributions for both the primary models and the repeated random cross-validation models by randomly shuffling the outcome variable. Supplementary Figure 2 depicts the sum of model weights by PFN for the primary ridge regression models in each of the matched samples.
